# Constitutional Flavonoids Derived from *Epimedium* Dose-Dependently Reduce Incidence of Steroid-Associated Osteonecrosis Not via Direct Action by Themselves on Potential Cellular Targets

**DOI:** 10.1371/journal.pone.0006419

**Published:** 2009-07-29

**Authors:** Ge Zhang, Xin-Luan Wang, Hui Sheng, Xin-Hui Xie, Yi-Xin He, Xin-Sheng Yao, Zi-Rong Li, Kwong-Man Lee, Wei He, Kwok-Sui Leung, Ling Qin

**Affiliations:** 1 Musculoskeletal Research Laboratory, Department of Orthopaedics & Traumatology, The Chinese University of Hong Kong, Hong Kong, China; 2 Institute of Traditional Chinese Medicine & Natural Products, College of Pharmacy, Jinan University, Guang Zhou, China; 3 Center for Osteonecrosis and Joint Preserving and Reconstruction, Department of Orthopaedic Surgery, Sino-Japan Friendship Hospital, Beijing, China; 4 Li Ka Shing Institute of Health Sciences, The Chinese University of Hong Kong, Hong Kong, China; 5 Department of Orthopaedics & Traumatology, The First Hospital Affiliated to Guang Zhou University of Traditional Chinese Medicine, Guang Zhou, China; 6 Department of Endocrinology and Metabolism, Shanghai Tenth People's Hospital, Tongji University, Shanghai, China; Purdue University, United States of America

## Abstract

Intravascular-thrombosis and extravascular-lipid-deposit are the two key pathogenic events considered to interrupt intraosseous blood supply during development of steroid-associated osteonecrosis (ON). However, there are no clinically employed agents capable of simultaneously targeting these two key pathogenic events. The present experimental study demonstrated that constitutional flavonoid glycosides derived from herb *Epimedium* (EF, composed of seven flavonoid compounds with common stem nuclear) exerted dose-dependent effect on inhibition of both thrombosis and lipid-deposition and accordingly reducing incidence of steroid-associated ON in rabbits, which was not via direct action by themselves rather by their common metabolite on potential cellular targets involved in the two pathogenic pathways. The underlying mechanism could be explained by counteracting endothelium injury and excessive adipogenesis. These findings encourage designing clinical trials to investigate potential of EF in prevention of steroid-associated ON.

## Introduction

Steroids are indicated for serious infectious diseases such as Severe Acute Respiratory Syndrome (SARS) and Acquired Immure Deficiency Syndrome, or for chronic autoimmune disease such as Systemic Lupus Erythematosus and Rheumatoid Arthritis. However, steroid-associated osteonecrosis (ON) frequently occurs. It is highly desirable to develop agents which could prevent ON occurrence due to its generally poor surgical prognosis [1]–[3].

The etiopathogenesis of steroid-associated ON has been recently explained by both intravascular thrombosis induced occlusion and extravascular lipid-deposit induced pressure, leading to impairment of intra-osseous blood supply [1], [3]–[4]. Endothelium injury, which predisposes to both hypercoagulation and hypofibrinolysis, has consistently presented itself in the intravascular events [5]; while elevated adipogenesis [6] is involved in extravascular events [7]. Although it has been experimentally confirmed that a combined administration of an anticoagulant with a lipid-lowering agent may help prevent steroid-associated ON [8], the ideal strategy would be simultaneously target both intravascular thrombosis and extravascular lipid deposition for preventing steroid-associated ON development [4].

**Figure 1:**
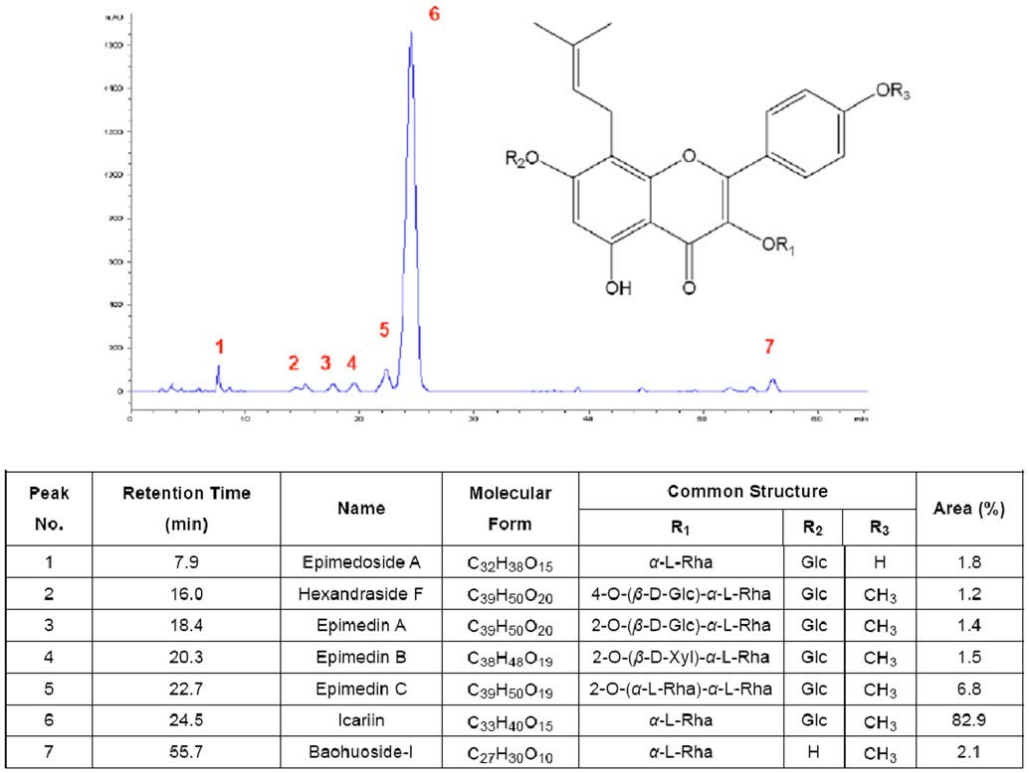
Seven major flavonoid compounds are identified in *Epimedium*-derived Flavonoids (EF). The common structure is 8-prenylkaempferol. R1 and R2 are substituted by glucose, rhamnose, or xylose, and R3 is replaced by methyl.

The authors' clinical epidemiological data showed that a lower prevalence (5–6%) of ON was found in patients recovered from SARS frequently prescribed with crude extract of flavonoids rich “Bone Strengthening Herb” *Epimedium* during their rehabilitation in southern China [9]–[10], whereas a higher prevalence (32.7%) [11] of ON was found in those seldom prescribed with crude extract of *Epimedium* in northern China. Recently, using small scale laboratory isolation procedure, “Bone Strengthening Herb” *Epimedium* derived flavonoids showed beneficial effect on prevention of steroid-associated ON with inhibition of both intravascular thrombosis and extravascular lipid-deposition in our established rabbit model with a single dose study design [7], [12]. Now, a simplified procedure for isolating flavonoids from herbal *Epimedium* to meet requirement of large scale production has been established (**International Application Number: PCT/CN2008/000165 issued by World Intellectual Property Organization**), which generates seven major flavonoid compounds with common stem nuclear characterized by high performance liquid chromatography (HPLC) profile (Figure 1). According to recent findings that diversiform isoflavones with common stem nuclei may be intestinally metabolized to Equal for acting on pharmacological targets [13], we predicted that all the seven flavonoid glycosides with common stem nuclei in the EF could be finally intestinally metabolized to a uniform molecule detected in serum.

Up to date, there were no available studies addressing dose-effect pattern and active substance basis of EF obtained from the simplified isolation procedure. Accordingly, the following two specific experiments were designed in the present study. Study I was to use our established rabbit model [7] to examine the effect of the EF in different dosage on haematological indices for intravascular thrombosis and extravascular lipid-deposition, as well as histopathological indices of ON lesion, intravascular thrombosis and extravascular lipid-deposition, respectively. Both prototype and metabolite of EF in serum were also examined by high performance liquid chromatography (HPLC)/ultra-violet (UV)/electrospray ionization-ion trap mass spectrometry (MS). Study II was to use human umbilical vein endothelial cell model and 3T3-L1 preadipocyte model in vitro to detect the prevention effect of Icaritin, the detectable metabolite of the EF, as well as those seven prototype flavonoid glycosides, on endotoxin-induced endothelial cell damage and steroid-induced lipid deposition, respectively.

## Materials and Methods

### Study I

#### Animal, Group and Treatment

One hundred and twelve male 28-week-old New-Zealand white rabbits with body weight of 4∼5 kg were housed at the Laboratary Animal Service Center of Prince of Wales Hospital, the affiliated hospital of the Chinese University of Hong Kong. The animals received a standard laboratory diet and water *ad lititum*. The experimental protocol was approved by Animal Experiment Ethics Committee of authros' institution in Hong Kong (Ref No. 04/038/MIS). Based on our established protocol for inducing steroid-associated ON [7], all the rabbits were intravenously injected with 10 µg. kg^−1^ of Lippolysaccharide (LPS; Escherichia coli 0111:B4, Sigma-Aldrich, Inc. USA) on day 0 (week 0). 24 hours later, three injections of 20 mg. kg^−1^ of Methylprednisolone (MPS; Pharmacia & Upjohn, USA) were given intramuscularly at a time interval of 24 hours. Simultaneously, the rabbits were divided into 4 groups for the following daily oral administration: low dose EF group (L-EF; n = 28; 10 mg. kg^−1^. day^−1^), middle dose EF group (M-EF; n = 28; 20 mg. kg^−1^. day^−1^), high dose EF group (H-EF; n = 28; 40 mg. kg^−1^. day^−1^), and control group received corresponding vehicle of EF daily (CON; n = 28).

#### Pre-euthanasia Evaluation

At week 0 (baseline, immediately before LPS injection), 1 and 2 post induction, 5 ml blood sample was collected. Plasma and serum were prepared for evaluating endothelium damage index TM (Thrombomodulin) using established protocols [7], [14]–[15] and detection of both prototype and metabolite of EF, respectively. In addition, serum hepatocyte injure indices, including Alanine aminitransperase (ALT) and Aspartate aminotransferase (AST), were examined for evaluating potential hepatocyte toxicity of EF. Marrow sample from iliac crest was also obtained for local evaluation of adipogenic potential index of mesenchymal stem cell (Adipocyte Positvie Colonies) [16]. On the other hand, dynamic-contrast-enhanced MRI was performed on proximal femur for intra-osseous perfusion function index (PEP, i.e. ‘peak enhancement percentage’) before sacrifice at baseline, week 1, and week 2 post-induction, using a bolus of dimeglumin gadopentetate (Magnevist; Schering, Berlin, Germany) with our established protocol [7], [12], [16].

Serum Examination for either Prototype or Metabolite of EF: Serum was obtained by centrifugation at 4500 revolutions per minute (rpm) for 15 min and stored at −80°C before analysis. All serum samples were thawed at room temperature. Analytes were extracted from 1 ml serum aliquots by liquid-liquid partition using water-saturated ethyl acetate. The ethyl acetate partitions were dried by gently blowing with nitrogen (N_2_) gas at room temperature. The residues were re-dissolved in 60% MeOH-H_2_O for injection into performance liquid chromatography-tandem mass spectrometry (HPLC-MS/MS) analysis, using an HPLC system coupled with a UV-DAD (Ultra-Violet) system and an ESI ion trap mass spectrometry. LC separation was conducted using an Agilent Series 1100HPLC system and a C18 reversed-phase (RP) column (5 µm, 4.6 mm×250 mm; Shimadzu, Japan) and 75% MeOH-H_2_O as mobile phase at a flow rate of 1.0 ml/min. MS analysis was performed using an Esquire 2000 ESI ion trap mass spectrometer (Bruker, USA) equipped with Esquire control software. Mass spectra were acquired and processed using the software provided by the manufacturer. The positive ion mode and MS/MS analysis were selected, working under the following conditions: Capillary Voltage = 4 kV; Capillary Temperature = 300°C; Nebulizer Gas Pressure = 30 psi; Drying Gas Flow Rate = 10 L/min. The scan range was m/z 50–1500, the maximum injection time was 50 ms, and two scan events were prescribed to run sequentially in the mass spectrometer.

Marrow Examination for Adipogenic Potential: Bone marrow was aspirated sterilely from either side of bilateral iliac crests of each rabbit using a pre-heparinized flexible plastic tube affixed to a 10-ml syringe containing 1 ml of heparin solution (1000 U/ml). Marrow mononuclear cells (MNCs) were isolated by discontinued gradient centrifugation on Ficoll (Density = 1.074 g/ml) at 1050 g for 25 minutes at room temperature. The isolated MNCs were cultured for mesenchymal stem cell (MSC) in basal medium, containing Dulbecco's modified Eagle's medium (DMEM), 10% fetal bovine serum (FBS), 1% mixture of penicillin, streptomycin and neomycin (Invitrogen 120 Corporation, Carlsbad, USA), which were incubated at 37°C, 5% humidified CO_2_. After three days, the medium was changed and nonadherent hematopoietic cells were discarded. Afterwards, the medium was changed once a week in two weeks. Two weeks later, the cells were seeded at a density of 5000 cells/cm^2^ for culture in the basal medium until 80% confluence in one week. Thereafter, the basal medium was repalced with an adipogenic medium (the basal medium supplemented with 15% normal horse serum and 100 nM dexamethasone) (Sigma, Demark) for culture in two weeks [17]. After 2 weeks in culture, the cells in vitro were stained with Oil red O. The colonies cotaining adipocytes that are positive for Oil red O were considered as adipocytic colonies. Percentage of Adipocytic Colonies per Total Colony Number (Adipocyte Positvie Colonies), as adipogenic potential index, was determined microscopically. A colony is defined as a distinct group of more than 16 cells and it represents the derect descedent of 1 MSC present at culture start [18]–[19].

#### Post-euthanasia Evaluation

4, 8 and 16 rabbits in each group were sacrificed at week 0, 1 and 2 post induction, respectively. The body weight of each rabbit before sacrifice was documented. Survival condition throughout the experiment period in each group was recorded.

Examination of ON Lesion: After sacrifice using overdose Sodium Pentobarbitone, the decalcified proximal femoral samples were embedded in Paraffin, cut into 6-µm-thick sections along the coronal plane for histopathological examination by Hematoxylin & Eosin (H&E) staining [7]. Identification of ON lesion was performed using established criteria, i.e. diffuse presence of empty lacunae or pyknotic nuclei of osteocytes in the trabeculae accompanied by surrounding necrotic bone marrow [20]–[21]. Rabbit that had at least one ON lesion in the areas was considered as ON^+^, while that with no ON lesion was considered as ON^−^. ON Incidence was defined as numbers of ON^+^ rabbits divided by numbers of total rabbits in each group, and ON Extent was defined as numbers of ON lesions per ON^+^ rabbit [7], [12].

Examination of Thrombotic Vessels Counts for Intravascular Thrombosis: Fifteen successive slices in each dissected proximal 1/3 part of bilateral femur samples of each rabbit were chosen for summation of histopathological index of intravascular thrombosis, i.e. Thrombotic Vessle Counts, usng a microscope imaging system (Zeiss Aixoplan with Spot RT digital camera, Zeiss, Germany) [7], [12].

Examination of Fat Cell Area Fraction for Extravascular Lipid-deposition: Fifteen successive slices in each dissected proximal 1/3 part of bilateral femur samples of each rabbit were calculated for average of Fat Cell Area Fraction, a histopathological index of extravascular lipid deposition, defined as total marrow fat cell area normalized by total marrow tissue area in each slice. The marrow fat cells with easily identified profile were quantified using a microscope imaging system (Zeiss Aixoplan with Spot RT digital camera, Zeiss, Germany) with image analysis software (ImageJ 1.32j, NIH, USA) [7], [12]. Intravascular thrombosis index (Thrombotic Vessle Counts), and extravascular lipid-deposition index (Fat Cell Area Fraction) were also histo morphometrically quantified.

### Study II

#### In Vitro Study using Human Umbilical Vein Endothelial Cell Damage Model

Human Umbilical Vein Endothelial Cells (HUVECs, SC-8000, ScienCell Research Laboratories, USA) were maintained in commercialized Endothelial Cell Medium (SC-1001, ScienCell Research Laboratories, USA). These HUVECs were incubated in a humidified atmosphere containing 5% CO_2_ at 37°C and passed every three days. HUVECs were cultured in 24-well plates at a density of 1∼2×10^5^/ml and allowed to grow to desired confluence. The cells were incubated with LPS (Escherichia coli 0111:B4, Sigma-Aldrich, Inc. USA) at a concentration of 0.625 µg/ml for 24 hours [22]. Before incubation with LPS, HUVECs were pretreated for 24 hours with Icaritin (HPLC Purity>99%, semi-synthesis by Shenzhen Research Institute of Tsinghua University, China) at 0 M (indcution group), 10^−16^ M (low dose group), 10^−14^ M (middle dose group), 10^−12^ M (high dose group) and parent compounds in EF (EF1 ∼ EF7) at 10^−14^ M. The control group was defined as non-LPS-incubated cells without Icaritin pretreatment but dimethyl sulfoxide (DMSO). Icaritin and LPS was dissolved using DMSO and the content of DMSO should never exceed 0.05% in all the groups. Based on an established protocol by manufacture, supernate soluble TM was measured using commercialized Human Thrombomodulin ELISA Kit (ab46508, Abcam, UK). All experiments were performed in triplicate.

#### In Vitro Study using Preadipocyte Lipid Deposition Model

NIH 3T3-L1 cells were grown in a phenol red-free Dulbecco's modified Eagle's medium (DMEM/F-12) (Gibco, USA) with 10% calf serum. Based on established adipogenic differentiation protocol with specific medium [23], Icaritin at 0 M (indcution group), 10^−16^ M (low dose group), 10^−14^ M (middle dose group), 10^−12^ M (high dose group) and parent compounds in EF (EF1 ∼ EF7) at 10^−14^ M was added to the medium over the full course of differentiation. The control group was defined as non-steroid-incubated cells without Icaritin treatment but DMSO. Icaritin and Dexamethasone was dissolved using DMSO and the content of DMSO should never exceed 0.05% in all the groups. Medium was changed every other day. One week later, the cells were washed with phosphate-buffered saline (PBS) twice, and then stained with 0.6% Oil Red O solution for 2 hours at room temperature. Stained Oil Red O was eluted with isopropanol after the cells were washed, and the optical density (OD) of the solution at 520 nm was measured [24]. All experiments were performed in triplicate.

#### Statistics

The ON Incidence was compared using Fisher's exact probability test. The cross-sectional quantificational data were expressed as Mean±SD, which were examined using Analysis of Covariance (ANCOVA) with body weight as covariant variable for eliminating its influence on the measurement results, using LSD' s post hoc multiple comparisons. The longitudinal quantification data were analyzed using ‘ANOVA of Repeated Measures’ with body weight as a covariant variable for eliminating its influence on the measurement results, using LSD' s post hoc multiple comparisons. The in vitro data was evaluated by ANOVA, using LSD' s post hoc multiple comparisons. All statistical analysis was performed using SPSS 10.0. Statistical significance for compaison was set at P<0.05.

## Results

### Prevention Efficacy and Safety Evaluation

**Figure 2:**
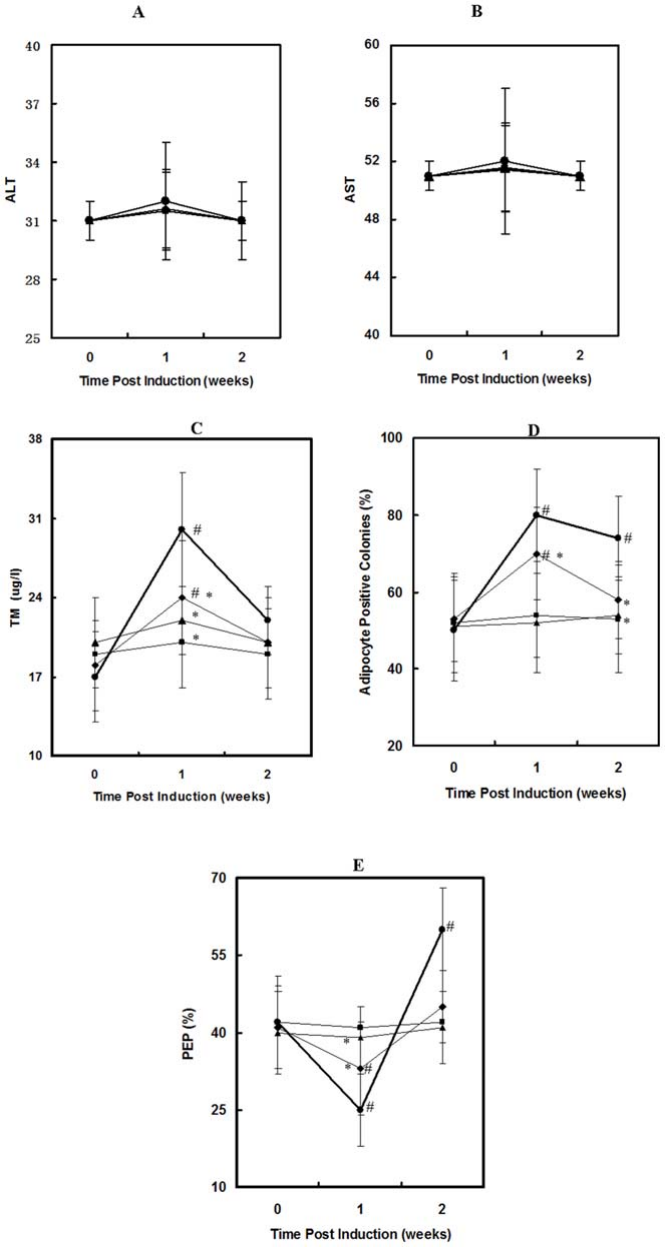
Hematology/cytology/MRI data analysis. There was no significant change from baseline in ALT (A) and AST (B) when compared to the CON group. Significantly increased TM (C) from baseline in the CON group was attenuated in the L-EF group or prevented in both the M-EF and H-EF group at week 1 post induction, and adipocyte positive colonies (D) in the CON group were attenuated in the L-EF group or prevented in both the M-EF and H-EF group after induction. In addition, the significantly decreased PEP (E) from baseline in the CON group was attenuated in the L-EF group or prevented in both the M-EF and H-EF group at week 1 post induction. Note: * P<0.05 for comparison with CON; # P<0.05 for comparison with baseline. • CON group; ⧫ L-EF group; ▪ M-EF group; ▴ H-EF group.

**Figure 3:**
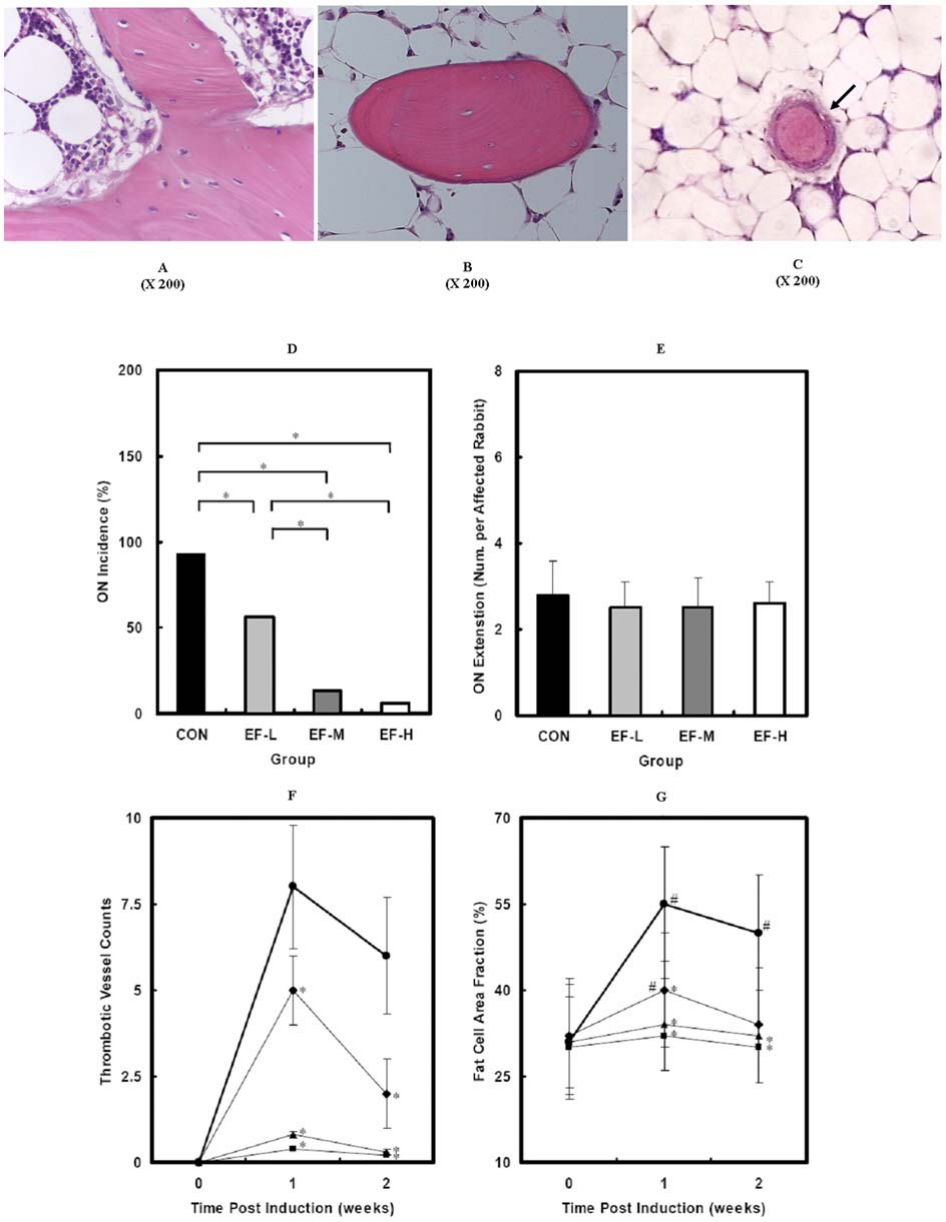
Key characteristics for histopathological identification and histopathological data analysis. ON lesion was found with trabecular bone containing considerable empty lacunae and lack of marrow cells (B) when compared to normal bone (A). In ON^+^ rabbits, thrombi were predominantly found in small marrow vessels with lack of angiographic particles (C), and marrow was predominantly occupied by a numerous fat cells (B, C). (D) Incidence of ON in each group: CON (13/14, 93%), L-EF (9/16, 56%), M-EF (2/16, 13%), H-EF (1/16, 6%). (E) There was no significant difference in ON Extent among all the groups. (F) Thrombotic Vessel Counts, and (G) Fat Cell Area Fraction presented similarities in changing patter over time, i.e. either attenuated in the L-EF group or prevented in both the M-EF and H-EF group when compared to that in the CON group. Note: Arrow pointed particle was angiographic substance during microCT-based angiography (data not shown). • CON group; ⧫ L-EF group; ▪ M-EF group; ▴ H-EF group; * P<0.05

Neither organ bleeding nor death was found in any group throughout the experimental period. Either ALT or AST, the index of hepatocyte injury, did not significantly change from baseline among all the groups except moderate increase at 1 week in the CON group throughout the experimental period (Figure 2A–B). ON lesion was not found until week 2 post induction in each group. ON lesion was histopathologically characterized with trabecular bone containing considerable empty lacunae (Figure 3A, 3B and 3C). The ON Incidence was 93% (15/16) in the CON group, 56% (9/16) in the L-EF group, 13% (2/16) in the M-EF group and 6% (1/16) in the H-EF group, respectively. Fisher's exact probability test showed that the ON incidence in the L-EF, M-EF and H-EF group was significantly lower than that in the CON group (P<0.05 for all). The ON Incidence in the M-EF and H-EF group was significantly lower than that in the L-EF group (P<0.05 for both), whereas no difference in the ON Incidence was found between the M-EF and H-EF group (P>0.05). There was no significant difference in the ON Extent among the CON group (2.8±0.8), L-EF group (2.5±0.6), M-EF group (2.4±0.7) and H-EF group (2.6±0.5) (Figure 3D and 3E).

### Effect on Pathogenic Events Involving Intravascular/Extravascular Abnormalities

Intravascular histopathology and its contributive events: Histopathologically, thrombi were predominantly found in small marrow vessels in the CON group since week 1 post induction (Figure 3B). The Thrombotic Vessel Counts in the CON group increased to peak level at week 1 post induciton, and then maintained until week 2 post indcution. There was a significant difference in the changing pattern between the CON group and the three EF groups. The increased Thrombotic Vessel Counts in the CON group were either attenuated in the L-EF group or prevented in both the M-EF and H-EF group throughout the experimental period. Fisher's exact probability test showed that the order of the Thrombotic Vessel Counts at week 1 post indctuion was smilar to those at week 2 post indctuion, i.e. CON>L-EF>M-EF = H-EF (Figure 3F). Mechanistically, TM in the CON group presented a significant increase at week 1 post induction (P = 0.000), then declined toward baseline. There was a significant difference in the changing pattern between the CON group and the three EF groups (P<0.05 for L-EF vs CON, P<0.01 for both M-EF vs CON and H-EF vs CON). Especially, the significant increase in TM in the CON group was either attenuated in the L-EF group or almost prevented in both the M-EF and H-EF group at week 1 post induction (Figure 2C).

Extravascular histopathology and its contributive events: Histopathologically, marrow was predominantly occupied by numerous fat cells in the CON group after induction (Figure 3C). The Fat Cell Area Fraction in the CON group increased signficantly at week 1 post induciton, and then maintained until week 2 post indcution (P<0.05 for both). There was a significant difference in the changing pattern between the CON group and the three EF groups. The significantly increased Fat Cell Area Fraction in the CON group was either attenuated in the L-EF group or prevented in both the M-EF and H-EF group throughout the experimental period. Fisher's exact probability test showed that the order of the Fat Cell Area Fraction at week 1 post indctuion was smilar to that at week 2 post indctuion, i.e. CON>L-EF>M-EF = H-EF (Figure 3G). Mechanistically, Adipocyte Positive Colonies in the CON group increased significantly at week 1 post induction (P<0.01), then moderately restored toward baseline. There was a significant difference in the changing pattern between the CON group and the three EF groups (P<0.05 for L-EF vs CON, P<0.01 for both M-EF vs CON and H-EF vs CON). Especially, the significant increase in the Adipocyte Positive Colonies was either attenuated in the L-EF group or almost prevented in both the M-EF and H-EF group at week 1 post induction (Figure 2D).

### Effect on Pathophysiology Reflecting Abnormality in Vascular Perfusion Function

The PEP in the CON group decreased significantly at week 1 post-induction (P<0.01), then significantly increased over the baseline at week 2 post-induction (P<0.01). There was a significant difference in the changing pattern of the PEP over time between the CON group and three EF groups (P<0.01 for L-EF vs CON, P<0.01 for both M-EF vs CON and H-EF vs CON). In particular, the significant decrease in the PEP in the CON group was attenuated in the L-EF group and almost prevented in both the M-EF and H-EF group at week 1 post induction (Figure 2E).

### Identification of Prototype and Metabolite of EF in Serum

**Figure 4:**
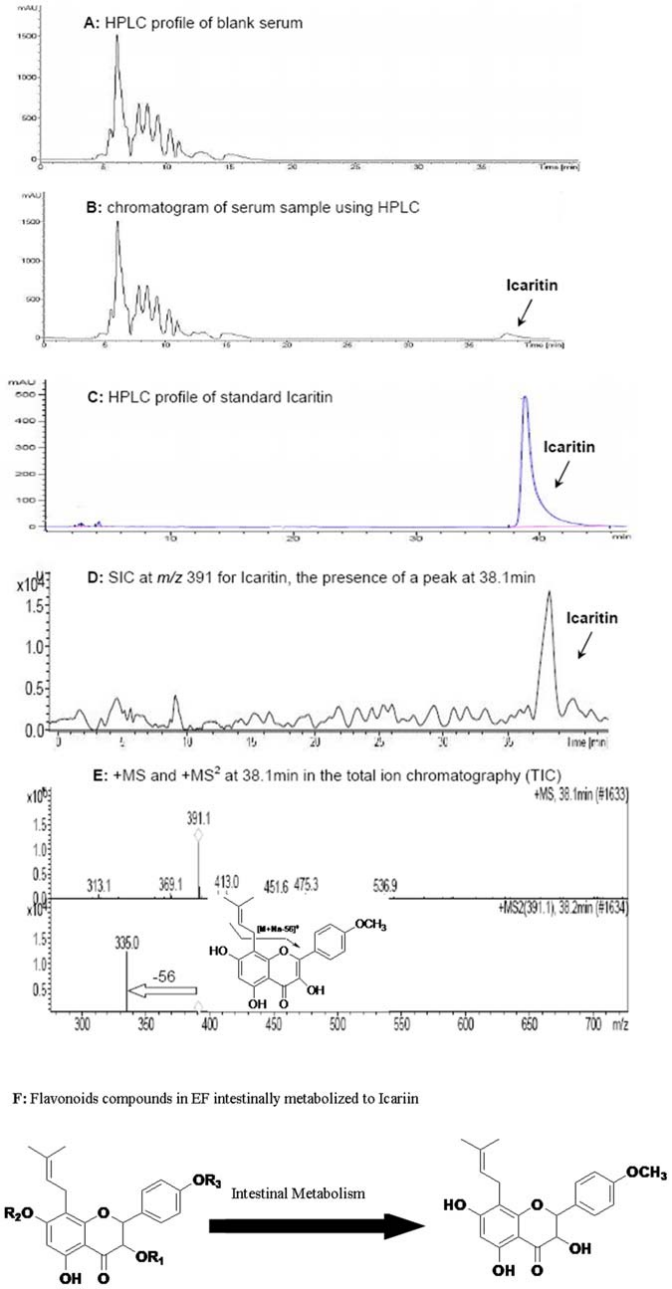
A total ion chromatogram in full scan mode generated by HPLC/UV/MS/MS. (A)∼(B) Compared with the blank sera, a peak shown in 38.1 min in the sera from L-EF, M-EF and H-EF group. (C) HPLC profile of standard Icaritin. (D) 391 (*m/z* [M+Na]^+^) for Icaritin selected for the subsequent selected ion chromatography (SIC), with a peak at 38.1 min. (E) The +MS showed the mass weight by 391 ion (*m/z* [M+Na]^+^) and the absence of 56 exhibited the existence of prenyl in the +MS^2^ chromatography. (F) *Epimedium*-derived flavonoids with common stem nuclei intestinally metabolized to Icaritin.

A total ion chromatogram in full scan mode was generated by HPLC/UV/MS/MS. Compared with the blank sera from the CON group, it was obviously that there was a peak in 38.1 min in the sera from three EF groups (Figure 4A∼B), which was structurally identified as Icaritin with the following steps. 1) Under the same mobile phase elution, the standard Icaritin was also washed out with the same retention time (Figure 4C); 2) 391 (*m/z* [M+Na]^+^) for Icaritin was selected for the subsequent selected ion chromatography (SIC), and a peak at 38.1 min was also present (Figure 4D); 3) Further, the +MS showed the mass weight by 391 ion (*m/z* [M+Na]^+^) and the absence of 56 exhibited the existence of prenyl in the +MS^2^ chromatography (Figure 4E), which firmly confirmed the structure of Icaritin. In addition, those seven flavonoid compounds were found not only absent in the HPLC profiles, but also not shown in the SIC profiles according to their mass weight.

### Effects of Icaritin (Serum Metabolite of EF) and seven prototype flavonoids in EF on Both Endothelial Cell Damage and Preadipocyte Lipid Deposition in vitro

**Figure 5:**
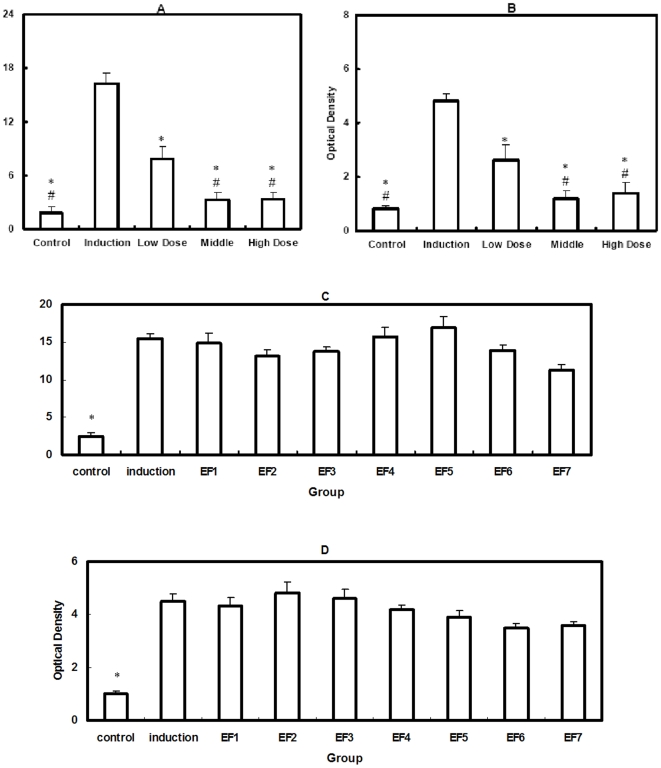
Supernate soluble TM in endotoxin induction group (A) and optical density in destained oil red O staining in steroid induction group (B) was significantly higher than the corresponding control group, respectively, whereas Icaritin dose-dependently lowered supernate soluble TM (A) and optical density in destained oil red O staining (B) when compared to the corresponding induction group, respectively. However, no differences were found between the seven parent flavonoids in EF at the concentration of 10^−14^ M and corresponding induction group both in supernate soluble TM (C) and optical density in destained oil red O staining (D). Note: * P<0.05 for comparison with the induction group. # P<0.05 for comparison with the low dose group.

In endothelial cell damage model, supernate soluble TM in endotoxin induction group was significantly higher than that in the control group (P<0.05), whereas Icaritin dose-dependently lowered supernate soluble TM at three different dosages when compared to the induction group (P<0.05 for all) (Figure 5A), but the seven prototype flavonoids in EF at the concentration of 10^−14^ M could not decrease the supernate soluble TM compared to the induction group (Figure 5C). In preadipocyte lipid deposition model, optical density in destained Oil Red O Staining in the induction group was significantly higher than that in the control group (P<0.05), whereas Icaritin dose-dependently lowered optical density at three different dosages when compared to the induction group (P<0.05 for all) (Figure 5B), but no differences were shown between the seven prototype flavonoids in EF at the concentration of 10^−14^ M and the induction group in optical density in Oil Red O staining (Figure 5C).

## Discussion

This was the first experimental study which demonstrated dose-dependent effect of *Epimedium*-derived flavonoids with common stem nuclear on inhibition of both intravascular thrombosis and extravascular lipid-deposition and accordingly reducing incidence of steroid-associated ON in rabbits.

### Dose-dependent Influence on Reducing Risk of Steroid-associated ON

The results of the calculated ON incidence indicated that EF was a potential phytotherapeutic agent for reducing risk of steroid-associated ON in a dose-dependent manner, which also provided experimental explanation for the previous clinical findings that ON prevalence was lower in patients recovered from SARS frequently prescribed with crude extract of *Epimedium* during their rehabilitation in southern China than those seldom prescribed with crude extract of *Epimedium* in northern China [9]–[11], [24]. On the other hand, no significant difference in the ON Extent among all the groups suggested a threshold beyond which development of ON lesion was initiated, which was consistent with the findings reported in our published study [12] and by other's experimental study [8]. It also indicated that once the threshold was reached, the prevention with EF had little effect on development of ON.

Vascular toxicity, e.g. organ bleeding, is a major concern on administration of a combination of an anticoagulant and a lipid-lowering agent in prevention of steroid-induced ON development even though such pharmaceutical combination strategy was experimentally confirmed for their efficacy [8]. The available experimental records of the present study indicated that EF was safe for vascular system throughout the observation period, which was evidenced by neither bleeding nor bleeding-related death under the employed three dosage regimens. In addition, hepatic safety is also an important concern. In the present study, a moderate (non-significant) increase in ALT or AST (hepatocyte injure index) was found in the CON group after induction with a combination of endotoxin and steroid. This is understandable since endotoxin has been reported to be detrimental to hepatocyte and steroid has been believed to lead to elevated lipogenesis and then overburden of lipid transportation of hepatocyte [4]. Further, ALT or AST did not significantly change from baseline when compared to the CON group among the three dosage groups, indicating hepatic safety of EF.

### Dose-dependent Influence on Inhibiting Extravascular Events

Histopathological examination suggested that EF was able to dose-dependently inhibit extravascular lipid deposition, which was evidenced by a significant increase in Fat Cell Area Fraction in the CON group while attenuated in the L-EF group or prevented in both the M-EF and H-EF group.

As to pathogenesis of extravascular lipid deposition during early stage of steroid-associated ON development, elevated adipogenesis was an important extravascular contribution event [25]. In terms of evaluation on condition of adipogenesis for this study, a high level of Adipocyte Positive Colonies (an adipogenic potential index) was considered to reflect excessive adipogenesis. In the present study, the results showed that excessive adipogenesis occurred at early stage of steroid-associated ON development, which was evidenced by the significantly increased level of Adipocyte Positive Colonies in the CON group at week 1 post induction. The experimental findings were also consistent with our previously published studies [7], [12]. Further, the results also indicated that EF was able to dose-dependently counteract elevated adipogenesis, which was evidenced by the findings that the significant increase in Adipocyte Positive Colonies in the CON group was attenuated in the L-EF group or almost prevented in both the M-EF and H-EF group at week 1 post induction.

### Dose-dependent Influence on Inhibiting Intravascular Events

The results suggested that EF was able to dose-dependently inhibit intravascular thrombosis, which was evidenced by the findings that the increased Thrombotic Vessel Counts in the CON group were attenuated in the L-EF group or prevented in both the M-EF and H-EF group throughout the experimental period.

As to pathogenesis of intravascular thrombosis, endothelium injury is recognized as an important initial contribution event in vascular diseases. Soluble TM in plasma is an index of endothelial injury. Endothelium TM is a cell surface glycoprotein that is primarily expressed in endothelial cells and, along with protein C, plays a role in counteracting both hypercoagulation and hypofibrinolysis after endothelial cell injury, which maintain balance between coagulation and fibrinolysis [26]. In the present study, a significant increase in TM in the CON group at week 1 post induction was found, which was also corresponding to a published study [27]. Further, our results also indicated that EF was able to dose-dependently protect endothelium from injuring, which was evidenced by the fact that the significantly increased TM in the CON group was either attenuated in the L-EF group or almost prevented in both the M-EF and H-EF group at week 1 post induction.

### Dose-dependent Influence on Preventing Decrease in Perfusion Function

A general consensus is that local vascular supply is the key to provide oxygen and nutrient to meet requirement of homeostatic tissue metabolism or repair [28]. PEP, as a parameter from dynamic contrast-enhanced MRI, may reflect ‘vascular quantity’ for vascular supply. In the present study, the significantly decreased PEP in the CON group at week 1 post-induction indicated insufficient vascular perfusion at an early stage. The results also indicated that EF was able to dose-dependently maintain ‘vascular quantity’ at an early stage, as evidenced by the findings, i.e. the significantly decreased PEP in the CON group was attenuated in the L-EF group and almost prevented in the H-EF group at week 1 post-induction.

### EF Exerted Prevention Effects not via Their Direct Action on Potential Target Cells

EF mainly consist of seven flavonoid glycosides with common stem nuclear. The question is how does EF exert their effect on cellular target? On one hand, Icaritin as a major intestinal metabolite, in the present study, was detected in the sera of the rabbits treated with EF, whereas those seven flavonoid glycosides were not detected, indicating that those seven flavonoid molecules in EF were intestinally metabolized to Icaritin (Figure 4F). This finding was also consistent with recent studies that Icaritin was the primary intestinal metabolite of prenyl flavonoids from *Epimedium* [29]. On the other hand, our unpublished data demonstrated neither measurable protection effect of EF on endotoxin-induced damage of human umbilical vein endothelial cell, nor measurable inhibition effect of EF on steroid-induced lipid deposition of 3T3-L1 Preadipocyte (data not shown). Further, the in vitro data from human umbilical vein endothelial cell model in the present study showed dose-dependent measurable protection effect of Icaritin on endotoxin-induced damage, which was evidenced by dose-dependently lowed supernate soluble TM in the Icaritin groups at three different dosages as compared to the induction group, however, no differences were found between the seven parent flavonoids in EF and the induction group in the supernate soluble TM. In addition, the in vitro data from 3T3-L1 Preadipocyte model in the present study showed dose-dependent measurable inhibition effect of Icaritin on steroid-induced lipid deposition, which was evidenced by dose-dependently lowed oil red O staining in the Icaritin groups at three different dosages as compared to the control group, but no differences were found between the seven flavonoids in EF and the induction group in the oil red O staining. The above two evidences were consistent with the HPLC/UV/MS/MS findings in study I, suggesting that EF compounds exerted their prevention effects not via direct action by themselves on potential cellular targets. In addition, our recently published animal study further demonstrated that Icaritin, the metabolite of EF, inhibited both thrombosis and lipid-deposition for reducing incidence of steroid-associated ON in a dose-dependent manner, implying that Icaritin might be a bioactive molecule served as common substance basis of those seven flavonoid glycosides with common stem nuclear in EF, though we have no sufficient evidence to conclude that Icaritin found in serum after EF administration was indeed causing the observed prevention effects of EF [16].

In the future, experimental study using steroid-associated ON model should be designed to test the hypothesis that EF inhibit both thrombosis and lipid-deposition for reducing incidence of steroid-associated osteonecrosis mainly via their common metabolite Icaritin. Glycosidase was thought to be one of the main enzymes to hydrolyze flavonoid glycosides to their corresponding aglycone in the intestinal, and gluconolactone, a broad specificity glycosidase inhibitor, can cause limited hydrolysis of flavonoid glycosides [30]. Thus, gluconolactone could be employed to limit the hydrolysis of EF, and as a result, prevent the formation of Icaritin from EF to see if the EF could reduce incidence of steroid-associated osteonecrosis with the existence of gluconolactone. If the hypothesis would be confirmed it will definitively not only facilitate understanding the basic biological sciences of chemistry metabolism of multi-component EF, but also help further explore molecular mechanism of EF for reducing risk of steroid-associated ON. Furthermore, a novel small molecule Icaritin would emerge into effective prevention of steroid-associated ON.

### Conclusion

Constitutional EF compounds exerted dose-dependent effect on inhibition of both thrombosis and lipid-deposition and accordingly reducing incidence of steroid-associated ON in rabbits, which was not via the direct action by themselves on potential cellular targets. The underlying mechanism could be explained by their counteraction effects on endothelium injury and excessive adipogenesis. These findings encourage designing clinical trials to investigate potential of EF in prevention of steroid-associated ON.
